# Antibacterial activities and action mode of anti-hyperlipidemic lomitapide against *Staphylococcus aureus*

**DOI:** 10.1186/s12866-022-02535-9

**Published:** 2022-04-26

**Authors:** Yufang Zhang, Yiying Zhang, Chengchun Chen, Hang Cheng, Xiangbin Deng, Duoyun Li, Bing Bai, Zhijian Yu, Qiwen Deng, Jie Guo, Zewen Wen

**Affiliations:** 1 Department of Infectious Diseases and Shenzhen Key Lab of Endogenous Infection, Shenzhen Nanshan People’s Hospital and the 6th affiliated Hospital of Guangdong Medical University, Shenzhen, 518052 China; 2 Quality Control Center of Hospital Infection Management of Shenzhen, Huazhong University of Science and Technology Union Shenzhen Hospital, Shenzhen, 518052 China; 3 Class of Biological Science, Futian District, Shenzhen College of International Education, No. 3 Antuoshan 6th Rd, Shenzhen, 518040 China

**Keywords:** *S. aureus*, Lomitapide, Biofilm formation, Quantitative proteomics

## Abstract

**Background:**

The increasing emergence of multidrug-resistant Gram-positive bacterial infections necessitates new antibacterial agents with novel mechanisms of action that can be used to treat these infections. Lomitapide has been approved by FDA for years in reducing levels of low-density lipoprotein (LDL) in cases of familial hypercholesterolemia, whereas the antibacterial effect of lomitapide remains elusive. In this study, the inhibitory activities of lomitapide against Gram-positive bacteria were the first time explored. Quantitative proteomics analysis was then applied to investigate the mechanisms of action of lomitapide.

**Results:**

The minimum inhibitory concentration (MIC) values of lomitapide against Gram-positive bacteria including both methicillin sensitive and resistant *Staphylococcus aureus*, *Staphylococcus epidermidis, Enterococcus faecalis*, *Enterococcus faecium*, and *Streptococcus agalactiae* were range 12.5–50 μM. Moreover, lomitapide also inhibited anti-biofilm activity against clinical *S. aureus* isolates. A total of 106 proteins with > 1.5-fold changes in expression were identified upon 1/2 × MIC lomitapide exposure, including 83 up-regulated proteins and 23 down-regulated proteins. Based on bioinformatics analysis, the expression of cell wall damage response proteins including two-component system VraS/VraR, lipoteichoic acid (LPA) D-alanylnation related proteins D-alanyl carrier protein (dltC) and carrier protein ligase (dltA), methionine sulfoxide reductases (mrsA1 and mrsB) were up-regulated. Moreover, the expression of SaeS and multiple fibrinogen-binding proteins (SAOUHSC_01110, FnBPB, SAOUHSC_02802, SdrC, SdrD) which were involved in the bacterial adhesion and biofilm formation, was inhibited by lomitapide. Furthermore, VraS/VraR deletion mutant (ΔvraSR) showed an enhanced lomitapide sensitivity phenotype.

**Conclusion:**

Lomitapide displayed broad antimicrobial activities against Gram-positive bacteria. The antibacterial effect of lomitapide may be caused by cell wall destruction, while the anti-biofilm activity may be related to the inhibition of surface proteins.

**Supplementary Information:**

The online version contains supplementary material available at 10.1186/s12866-022-02535-9.

## Background

*Staphylococcus aureus* is a gram-positive bacterium commonly colonized on human skin and the nasopharynx of healthy adults. Once entered into the internal tissues or bloodstream of the human host, it may result in a wide-spectrum of dangerous infectious diseases, such as skin and soft tissue infections, gastroenteritis, bacteremia, endocarditis, and pneumonia [1, 2]. *S. aureus* remains one of the predominant causes of community-acquired or hospital-acquired infection worldwide, as well as a top lethal factor in treatment due to its common antibiotic resistance [3]. Clinical *S. aureus* isolates have obtained resistance against most beta-lactam antibiotics, most commonly resistant to penicillin and methicillin [4, 5]. Recently, *S. aureus* with the resistance toward the last-resorted antibiotics, such as linezolid, vancomycin, and daptomycin, has also been increasingly reported [6–8]. The emergence of the multi-drug resistance *S. aureus* complicates treatment by increasing cost and length of stay [9], which drives us to develop novel antimicrobial agents for the treatment of *S. aureus*.

The biofilm formation ability of *S. aureus* is another challenge to its clinical treatment [10]. *S. aureus* biofilm is formed by secreting extracellular macromolecules mainly composed of lipid, protein, and a small amount of sugar. The biofilm allows bacterial cells to cling to surfaces of indwelling medical equipment, such as artificial valves and cardiac pacemakers, leading to chronic infections [11]. What’s more, multiple studies have shown that bacterial cells embedded within the biofilm are able to withstand higher concentrations of antibiotics than planktonic cells, making them harder to be killed or removed [12, 13]. Therefore, it is necessary for clinicians to put attention to the effect of drugs against bacterial biofilm when searching for novel choices to treat *S. aureus* infection [14, 15].

**Fig. 1:**
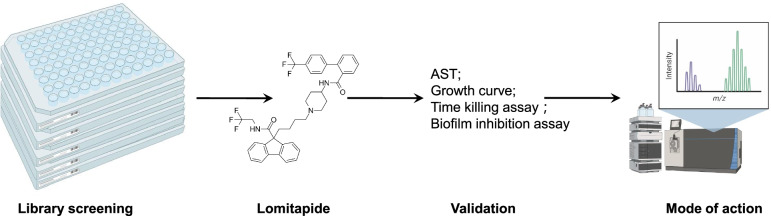
A flow chart diagram Showing the procedures of this study and the molecular structure of lomitapide. AST antimicrobial susceptibility testing

A cost-effective and promising strategy for exploring novel antibiotics is to re-explore formerly approved clinical drugs for antibacterial effects, as they were already tested on humans with relatively complete information about their toxicity and pharmacology [16, 17]. The successes of repurposed drugs have been proved in other fields such as oncology and cardiovascular diseases [18, 19]. Approved by the FDA in 2013, lomitapide has long been used as a treatment for homozygous familial hypercholesterolemia, with the highest dosage of 60 mg/day after tolerance is established [20]. Lomitapide functions in the human body by inhibiting a crucial part in cholesterol formation, the microsomal triglyceride transfer proteins (MTP), so to impede lipoprotein production and decrease LDL cholesterol levels. In this paper, we report the broad-spectrum inhibitory activities of lomitapide against clinical Gram-positive bacteria including *S. aureus*, *S. epidermidis, E. faecalis*, *E. faecium*, and *S. agalactiae*, as well as its impacts on the biofilm formation of *S. aureus*. Furthermore, mechanisms of antibacterial action of lomitapide were explored by quantitative proteomics. For clarity, the detailed steps employed in this study is represented as a flowchart [21] (Fig. 1).

## Results

### Lomitapide exhibited antibacterial activity against Gram-positive bacteria

In our screen of the FDA-approved drugs, we found that lomitapide exhibited inhibitory activity on standard strains ATCC29212 and ATCC29213. Further, the MICs of 49 *S. aureus* strains (48 clinical isolates and one standard strain) were measured and the results were listed in Table 1. MICs values of lomitapide for all *S. aureus* clinical strains were ranged between 12.5 μM and 25 μM, including these intermediate to daptomycin and linezolid (Table S1). Moreover, MICs in 81.8% of MRSA clinical isolates were showed with 25 μM and that in 81.3% of MSSA isolates were 12.5 μM, indicating that MRSA may have lower antimicrobial susceptibility than MSSA toward lomitapide. In addition, MICs of lomitapide against clinical *S. epidermidis, E. faecalis*, *E. faecium*, and *S. agalactiae* were also measured, as showed in Table S2*.* The MIC_50_ and MIC_90_ for the clinical Gram-positive bacteria were both 25 μM. Moreover, MICs for all clinical strains were showed with ≤ 50 μM, suggesting that lomitapide showed broad-spectrum antibacterial activity against Gram-positive bacteria. However, no inhibitory activity was observed against Gram-negative bacteria (Table S3).

**Table 1:** The MIC distribution of lomitapide against 49 clinical *S. aureus* strains

*S. aureus*	No. of isolates tested	No. (%) of isolates		MIC_50 _(μM)	MIC_90 _(μM)	MIC Range
		LTP MIC = 12.5 μM	LTP MIC = 25 μM			(μM)
MRSA	33	6 (18.2%)	27 (81.8%)	25	25	12.5–25
MSSA	16	13 (81.3%)	3 (18.7%)	12.5	25	12.5–25
Total	49	19 (38.8%)	30 (61.2%)	25	25	12.5–25

*LTP* Lomitapide, *MIC* Minimum inhibitory concentration

**Fig. 2:**
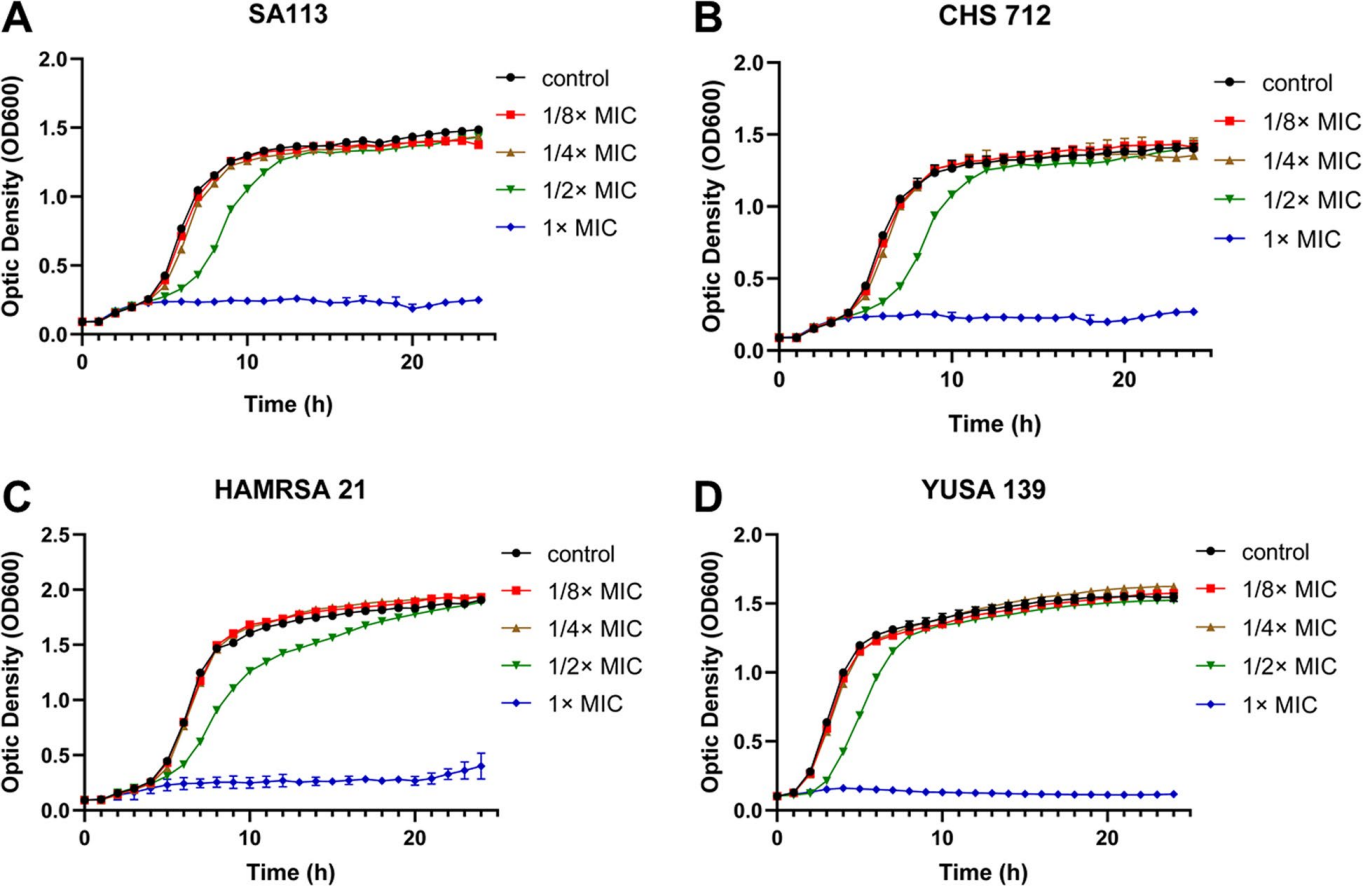
The inhibitory effect of lomitapide against the *S.aureus* planktonic growth. The planktonic growth of four *S. aureus* strains were cultured separately and measured under various concentrations of Lomitapide, including 1 × MIC, 1/2 × MIC, 1/4 × MIC, 1/8 × MIC, in SA113 (**A**), CHS712 (**B**), HAMRSA21 (**C**) and YUSA139 (**D**). CHS712, YUSA139 and HAMRSA21 were MRSA clinical isolates. MIC for all strains in this experiment were showed with 25 μM. Data are presented as means ± SD

Three clinical isolates, including YUSA139, HAMRSA21, CHS712, and standard strain SA113 were used to determine the inhibitory effect of lomitapide at sub-inhibitory concentrations (from 1/8 × MIC to 1 × MIC) against the planktonic cell growth of *S. aureus*. The data have been shown in Fig. 2, suggesting that *S. aureus* growth was completely suppressed at 1 × MIC, and obvious inhibition effects were showed at 1/2 × MIC, although did not affect the biomass of stationary stage. Furthermore, just slight or no inhibitory effect of lomitapide at 1/4 × MIC and 1/8 × MIC against *S. aureus* was showed in the control group.

The ability and efficiency of Lomitapide to kill *S. aureus* bacteria was tested and compared with vancomycin by time killing assay. Our data indicated that vancomycin showed its rapid bactericidal effect and killed the most bacteria after the exposure for 3 h at 8 × MIC. By contrast, lomitapide displayed the minimal bactericidal effect at 2 × and 4 × MIC and further showed the slight bactericidal effect at 8 × MIC (Fig. S1). This suggests that lomitapide is bacteriostatic rather than bactericidal against *S. aureus.*

### Lomitapide inhibited *S. aureus* biofilm formation

**Fig. 3:**
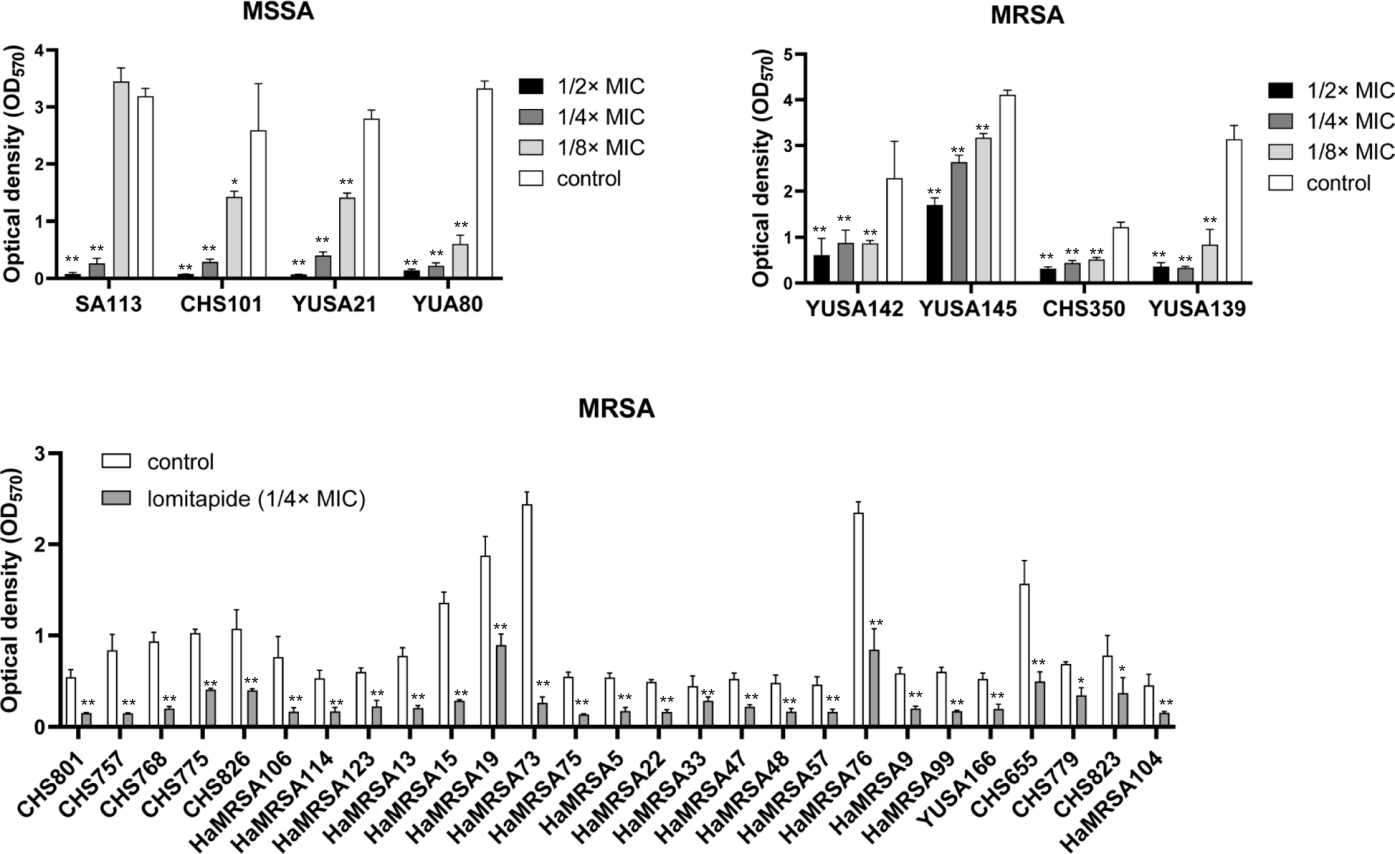
The anti-biofilm activity of lomitapide against *S. aureus*. **A** and **B** Four MRSA isolates (YUSA142, YUSA145, CHS350 and YUSA139), four MSSA isolates (YUSA80, SA113, YUSA21 and CHS101) were tested for the inhibitory effect of lomitapide on the biofilm formation under sub-inhibitory concentrations. **C** The anti-biofilm effect of lomitapide against 27 clinical MRSA isolates was assessed at 1/4 × MIC. The biofilm formation after 24 h incubation was measured by optic density (OD_570_) after dyeing with crystal violet. Data are presented as means ± SD. *: *p* < 0.05; **: *p* < 0.01

The inhibitory effect of lomitapide on *S. aureus* biofilm formation was then determined. The biofilm biomass was quantified by crystal violet staining. Lomitapide at sub-inhibitory concentrations ranging from 1/2 × MIC to 1/8 × MIC were added to the isolates, and biofilm formation was determined after 24 h at OD_570_. As showed in Fig. 3A and Fig. 3B, lomitapide significantly inhibited the biofilm formation of all MSSA and MRSA isolates at concentrations of 1/4 × MIC and 1/2 × MIC. Furthermore, The inhibitory activity of lomitapide on the biofilm formation of *S. aureus* was further investigated in 27 clinical *S. aureus* isolates. A significant decrease of biofilm could be observed after 24 h at all tested clinical *S. aureus* isolates (Fig. 3C), demonstrating the anti-biofilm capacity of lomitapide against clinical *S. aureus*.

### Quantitative proteomics analysis

**Fig. 4:**
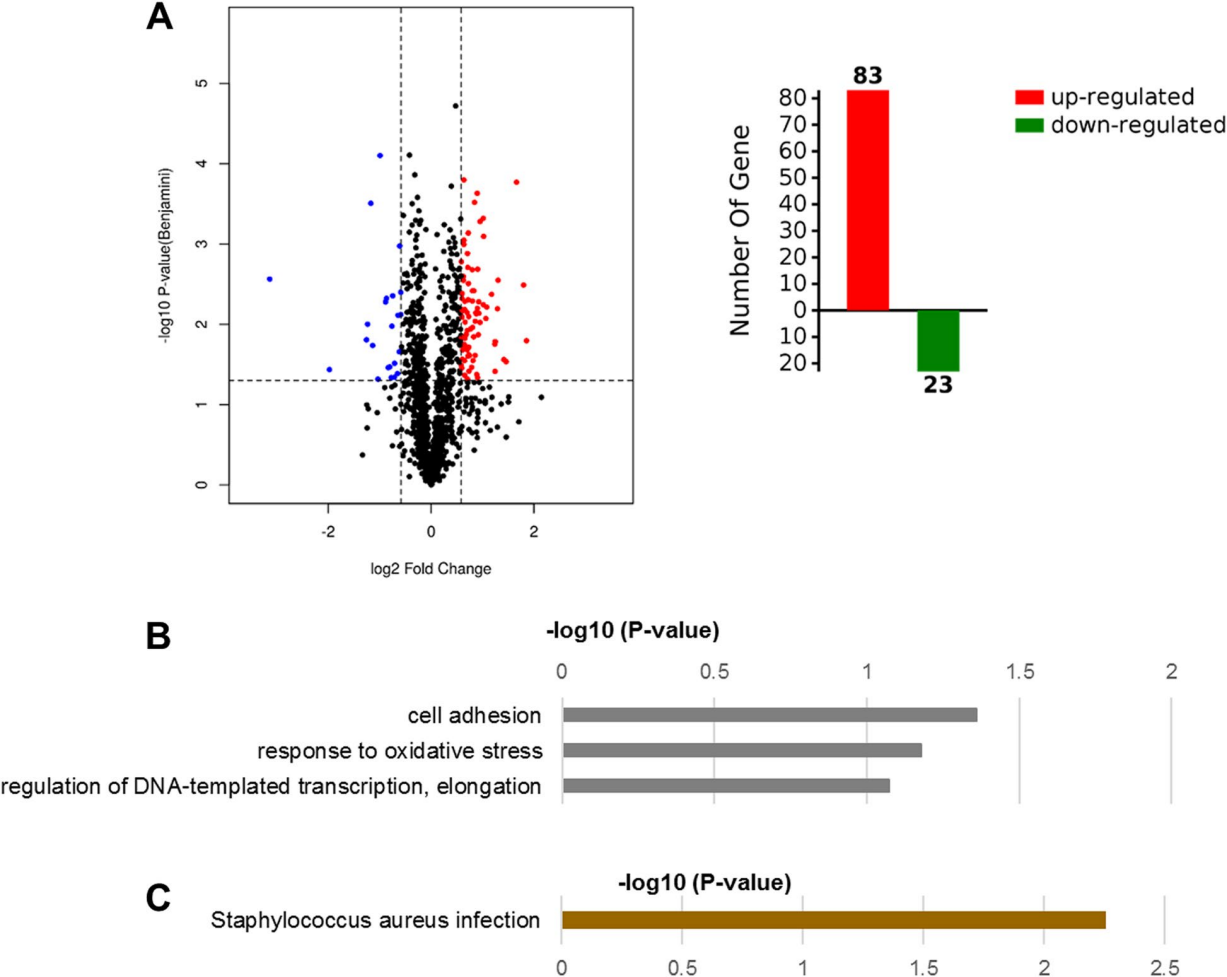
Differentially expressed proteins between the control groups and lomitapide-treated (at 1/2 × MIC) groups found by proteomics analysis. **A** Volcano map and total number of differentially expressed proteins, the horizontal axis represents the ratio of differentially expressed proteins in lomitapide treated group and untreated group of *S. aureus*, red shows an increase after treated and blue shows decrease. Vertical axis represents p-value between the two groups. **B** Gene Ontology analysis applied to differentially expressed proteins according to biological process. **C** KEGG analysis of the differentially expressed proteins using the DAVID database [22]

Quantitative label-free proteomic analysis was performed to investigate the impacts of lomitapide on the global proteomic response of the *S. aureus*. The expression profiles of proteins of clinical MRSA strain YUSA139 treated with 1/2 × MIC (12.5 μM) of lomitapide for 2 h were conducted for the quantitative proteomics analysis. Totally, 1,430 proteins (matched peptides ≥ 1, and FDR < 0.01) with 81,502 unique peptides were identified. Among the proteins quantified, the expression level of 106 proteins showed significantly different expression levels ( ≥|1.5|-fold change, *p* ≤ 0.05), 83 proteins were up-regulated, and 23 proteins were down-regulated in comparison to the control group (Fig. 4A and Table S4). Gene Ontology (GO) enrichment analysis was then performed to analyze the biological processes and molecular functions of the differentially expressed proteins found between these two groups. For biological processes, terms of cell adhesion, response to oxidative stress, and regulation of DNA-templated transcription, elongation were significantly enriched (Fig. 4B). The Kyoto Encyclopedia of Genes and Genomes (KEGG) pathway enrichment analysis showed that differentially expressed proteins involved in virulence pathways of *S. aureus* infection were significantly enriched in the lomitapide treatment group (Fig. 4C). The most significantly enriched terms and differentially expressed proteins involved within each are showed in Table S5.

**Fig. 5:**
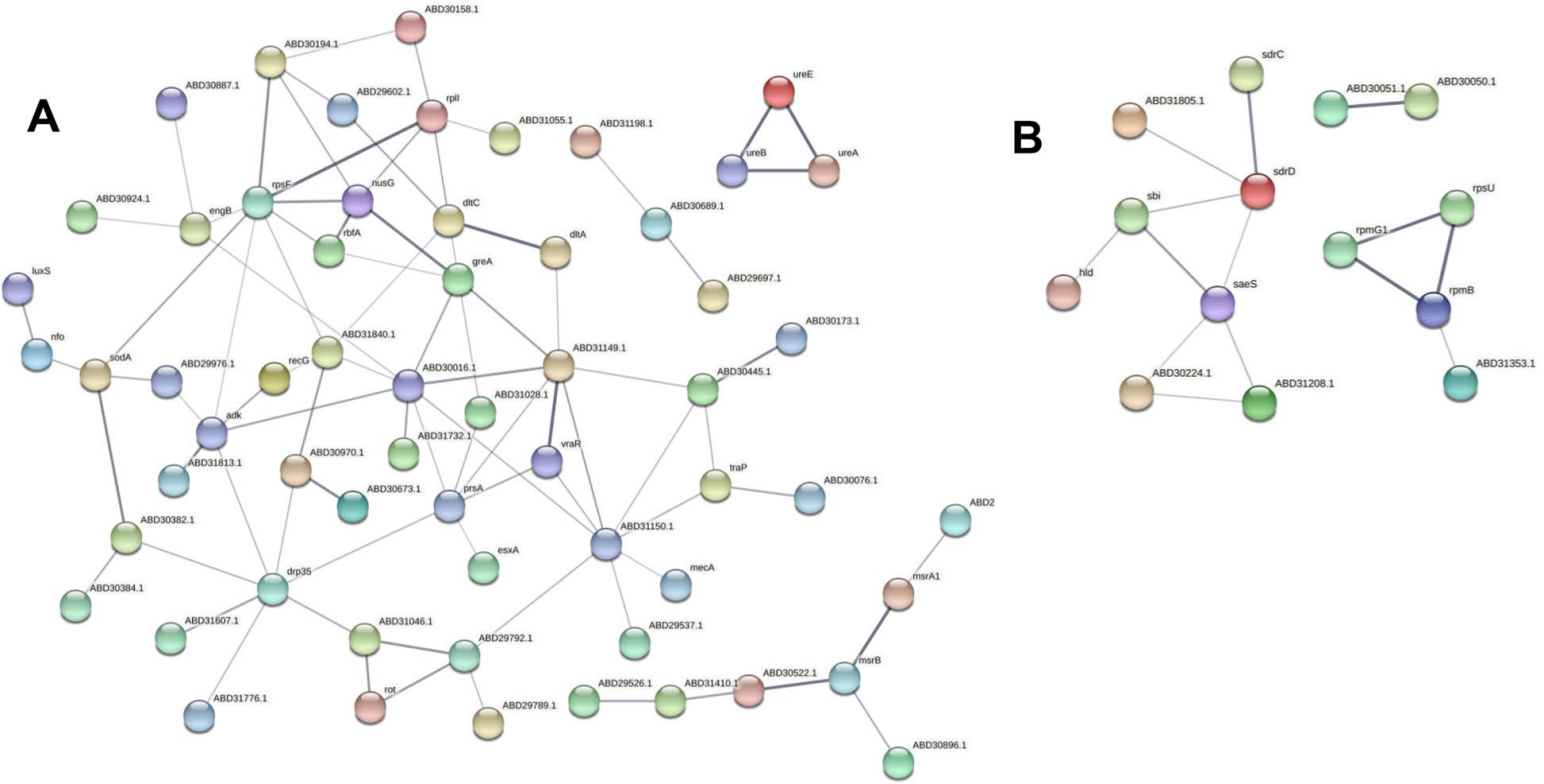
Protein − protein interaction network analysis for up-regulated proteins (**A**) and down-regulated proteins (**B**) after treated with 1/2 × MIC lomitapide base on STING database. Line thickness indicates the strength of data support. The disconnected nodes in the network are hidden

The interactions between proteins are showed by the Protein–protein interaction (PPI) network, created by STRING at confidence score = 0.400. Overall, the top hub of up-regulated proteins with the highest degree of connectivity in the PPI network was enrichment in protein biosynthesis, two-component regulatory system VraS/VraR, D-alanine-D-alanyl carrier protein ligase dltA, and D-alanyl carrier protein dltC involved in the D-alanylation of lipoteichoic acid (LTA), and urease (Fig. 5A). the top hub of down-regulated proteins was enrichment in ribosomal protein and two-component regulatory system SaeS involved in the regulation of staphylococcal virulence factors (Fig. 5B). Multiple proteins regulated by SaeS are inhibited by lomitapide, including fibrinogen-binding protein SAOUHSC_01110, fibronectin binding protein B SAOUHSC_02802, MHC class II analog protein SAOUHSC_02161, fibrinogen-binding protein SdrC, fibrinogen-binding protein SdrD, Immunoglobulin-binding protein Sbi, and Delta-hemolysin.

### The two-component regulatory system VraS/VraR was involved in the antibacterial activity of lomitapide

**Fig. 6:**
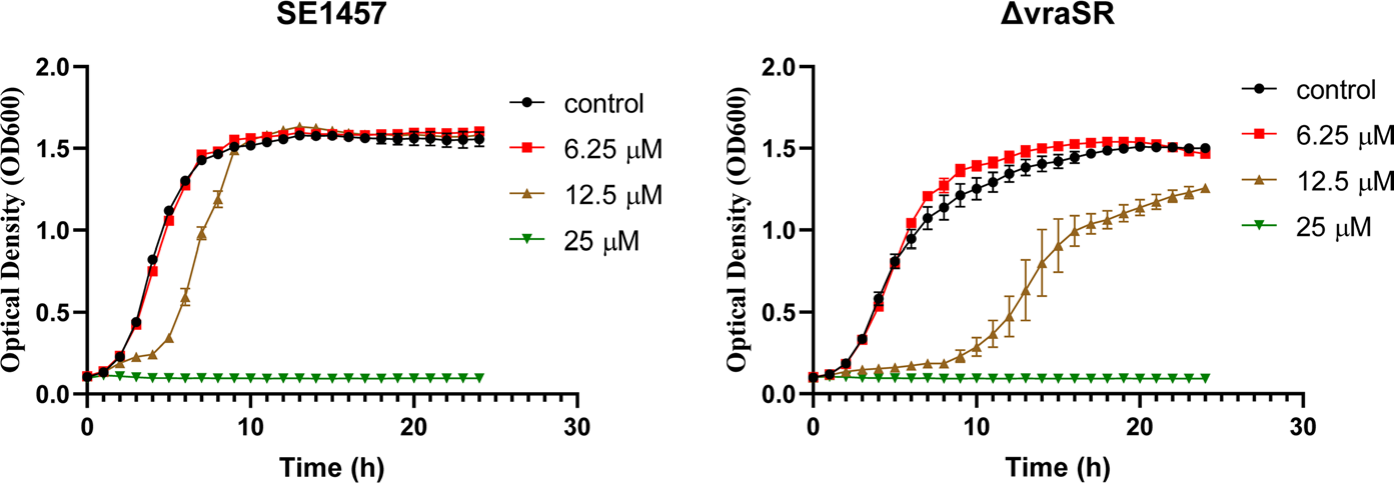
Growth curves of SE1457 and VraS/VraR deletion mutant (ΔvraSR) with lomitapide. The planktonic growth of *S. epidermidis* parent strain SE1457 and VraS/VraR deletion mutant (ΔvraSR) were monitored under various concentrations of lomitapide, including 6.25 μM, 12.5 μM, and 25 μM. Data are presented as means ± SD

To further validate the correlation of the VraS/VraR two-component regulatory system and the antibacterial activity of lomitapide, the susceptibility of VraS/VraR deletion strain　(ΔvraSR) of *S. epidermidis* was examined by growth curve [23]. As showed in Fig. 6, compared with that in the parent strain SE1457, the exponential growth phase of ΔvraSR appeared late after exposed to 12.5 μM lomitapide. In addition, the maximum growth level of ΔvraSR was also smaller than that of the parent strain SE1457 after cultured for 24 h, suggesting that the VraS/VraR two-component regulatory system participated in the antibacterial activity of lomitapide.

## Discussion

In this study, lomitapide was fristly proven to exhibited broad antibacterial activity against gram positive bacteria in vitro, including *S. aureus*, *S. epidermidis*, *E. faecalis*, *E. faecium*, and *S. agalactiae*. This study focused on the antibacterial effect of lomitapide on *S. aureus*, both MSSA and MRSA. Although the MICs of lomitapide are relatively higher than last-resort antibiotics such as daptomycin, vancomycin and linezolid, however, it has been long approved by FDA for treating familial hypercholesterolemia and reducing LDL level, meaning the advantage of guaranteed safety and being backed up by a large amount of clinical data. Nevertheless, its potential to be used as a clinical antibiotic needs further assessment considering the dosage of lomitapide and its in vivo drug concentrations. Lomitapide not only demonstrated an inhibitory effect on planktonic cells, but also on the biofilm formation of *S. aureus.* Biofilm formation often causes chronic infection and impedes clinical treatment by extending the length of stay, resulting in increased expenses and high mortality [10, 11]. In this study, lomitapide has shown strong biofilm formation inhibition at 1/2 × MIC and 1/4 × MIC against clinical *S. aureus* isolates. Therefore, we supposed that lomitapide could potentially be used for anti-infection treatment against *S. aureus* infections. Meanwhile, further work would need to be done to find lomitapide analogs with enhanced antibacterial effects.

Mode of action studies has great significance in improving the potency of antibacterial agents. With the development of mass spectrometry (MS) technologies, advanced MS-based proteomics has been extensively applied to provide a comprehensive understanding of the global response to bacteria to antimicrobial agents, which will help illuminate the modes of action. Global proteomics response of *S. aureus* to lomitapide reflects a comprehensive view of the bacterial cellular response under lomitapide stress, and the identified proteins affected are an important step toward unraveling its mechanism of efficacy. Following proteomics data revealed that the two-component regulatory system VraS/VraR members were both induced by lomitapide. The VraS/VraR was the key regulatory system that modulates the stress response of *S. aureus* elicited upon exposure to cell wall antibiotics including vancomycin, daptomycin, glycopeptides and β-lactams, which referred to as the cell wall stress stimulon [24–26]. In line with that, D-alanyl carrier protein (dltC) and carrier protein ligase (dltA), associated with lipoteichoic acid (LPA) D-alanylnation, which has an important role in modulating properties of the cell wall, were both increased after being treated with lomitapide [27]. LPA was commonly found in Gram-positive bacteria as a molecular composition of the cell wall or cell membrane [28]. Biosynthesis of LPA was catalyzed by the two proteins where dltA facilitates the transfer of D-alanine onto its peptidyl carrier protein dltC [27, 29]. The carrier protein then carries it to other proteins on the pathway (supposedly dltB) to transport it across the membrane and modify it into LPA and wall teichoic acids [30]. Furthermore, the enzymes involved in the D-alanine modification have been identified as a potential target for the development of novel antibacterial agents [31]. In addition, the repair enzymes methionine sulfoxide reductases (mrsA1 and mrsB), which are usually found as a result of cell wall active antibiotics [32], were up-regulated by lomitapide. Further, deletion of VraS/VraR significantly increased the susceptibility of *S. epidermidis* against lomitapide. Taken together, all these stress responses suggested that lomitapide may act on the cell wall of *S. aureus*. Interestingly, this possible target of the pathway is very similar to that of vancomycin, which acts by binding with D-alanyl-D-alanine (D-ala-D-ala) and prevents its contact with the transglycosylase, leading to failure in the assembly of the cell wall [33].

Another significant change of *S. aureus* proteome after lomitapide treatment was that the expression of SaeS various virulence factors was inhibited, such as fibrinogen-binding protein SAOUHSC_01110, FnBPB, MHC class II analog protein SAOUHSC_02161, SdrC, SdrD, Immunoglobulin-binding protein Sbi and Delta-hemolysin. The SaeRS two component system plays a major role in controlling the production of multiple virulence factors including hemolysins, leukocidins, superantigens, surface proteins, and proteases. *S. aureus* fibrinogen-binding proteins belong to the microbial surface component recognizing adhesive matrix molecules, which were involved in bacterial adhesion and virulence [34]. Furthermore, SdrC and the fibronectin/fibrinogen-binding proteins have been demonstrated to be implicated in biofilm matrix formation [35–37]. For instance, SdrC was engaged in low-affinity homophilic bonds that promote cell–cell adhesion, as well as mediates strong cellular interactions with hydrophobic surfaces when biofilm was initial attaching and growing [35]. Besides, previous studies showed that sdrC and sdrD are contributing to its attachment on human nasal epithelial cells and on medical equipment [38, 39]. The down-regulation of these proteins after being treated by lomitapide could be the potential reason for this drug’s inhibitory effect against *S. aureus* biofilm formation, which could be potentially applied to treat chronic *S. aureus* infections and reduce its transmission capabilities in clinic settings. In addition, since these cell surface proteins and Delta-hemolysin are also related to the *S. aureus* invasiveness, immune response evasion and hemolysis [40], it is speculated that lomitapide could also be used to reduce the virulence of *S. aureus*.

In conclusion, this study first found lomitapide to have antibacterial activities against *S. aureus* (both MSSA and MRSA) to planktonic cell growth and extracellular biofilm formation. The molecular mechanism was examined by proteomics analysis and points to some possible directions, including LPA synthesis onto the cell wall. Future investigations could dig deeper into the mode of action of lomitapide by looking for its antibacterial targets.

## Methods

### Bacteria strains and chemicals

A total of 49 clinical isolates of both MRSA and MSSA, 10 clinical strains of *E. faecalis*, *E. faecium*, and *S. agalactiae* in this study, were retrospectively collected between January 1, 2015 and December 31, 2018 at a general tertiary hospital in Shenzhen (Guangdong District, China). The genus and species of the isolates were identified by VITEK 2 compact system (Biomérieux, Marcy l’Etoile, France). Standard strain *E. faecali*s ATCC29212 and *S. aureus* ATCC29213 were kept in our laboratory and used as the representative control. All procedures involving human participants were carried out in accordance with the ethical standards of Shenzhen Nanshan People's Hospital. No formal consent is required for this type of study. Lomitapide was purchased from MedChemExpress (MCE, Shanghai, China).

### Antimicrobial susceptibility testing (AST)

The MICs of vancomycin, daptomycin, linezolid and lomitapide against *S. aureus. E. faecalis, E. faecium,* and *S. agalactiae* were measured by the broth dilution method [41]. Overnight cultures were adjusted to 0.5 McFarland turbidity, then diluted at 1:200 proportion with cation-adjusted Mueller Hinton broth (CAMHB, Huankai, Guangdong, China). Cultures experience a 24 h incubation process in 96-well plates with descending concentrations of drug. The MIC is defined as the lowest concentration of drug that inhibited the visible growth of bacteria. All of the MIC results of antibiotics were referred according to Clinical and Laboratory Standards Institute (CLSI) breakpoints.

### Growth curve assay

Overnight cultures of *S. aureus* were diluted 1:2000 with TSB (Tryptone soy broth, Huankai, Guangdong, China) and added lomitapide until reached concentrations of 1 × , 1/2 × , 1/4 × , and 1/8 × MIC. A control group without any drug being added was then established to make a comparison. They were cultured at 37 °C and shaken at 200 rpm, optical density at 600 nm (OD_600_) was measured for each well every 60 min for 24 h to determine planktonic cell growth. Growth curves were expressed in terms of OD_600_.

### Time kill studies

The bactericidal activities of lomitapide on the planktonic cells were determined by time-kill studies. *S. aureus* MRSA isolate YUSA145 with lomitapide MIC of 25 μM were selected, concentrations of 2 × MIC, 4 × MIC or 8 × MIC lomitapide were tested. Lomitapide was added to make the final concentration when the YUSA145 cultured at the logarithmic phase. After exposure for 0, 2, 6, and 24 h, 100 μL bacterial cultures was taken out and serially diluted with Mueller–Hinton broth, 5 μL aliquots was plated onto Mueller–Hinton agar. The viable cell was monitored by the CFUs counted after incubated 24 h at 37◦C.

### Biofilm inhibition assay

Biomass of the biofilm formed by *S. aureus* was determined by optical density of 570 nm (OD_570_) after dyeing with crystal violet (Thermo Fisher Scientific, Ohio, USA) staining according to the previous study [42]. This assay was conducted in 96-well plates by adding 100 uL of overnight cultures of *S. aureus*, 1:200 diluted with TSBG (Tryptone soy broth with 2% glucose) added to stabilize the biofilm, with 100 uL of descending concentration of lomitapide. Concentrations of lomitapide at 1/2 × , 1/4 × , and 1/8 × MIC were used, as well as a control with TSBG only. *S. aureus* strains were cultivated for 24 h and then dyed with crystal violet staining to color the biofilm formed. OD_570_ were then measured and recorded to determine the biomass of biofilm grown, OD_600_ were measured as well to determine the growth of planktonic cells.

### Sample preparation for quantitative proteomics analysis

Clinically isolated MRSA strain YUSA139 was inoculated and cultured to exponential growth phase (OD_600_ at 0.5) in TSB and divided into two groups with three replicates, the control group and the drug treated group. Lomitapide was added to the drug treated group until a final concentration of 1/2 × MIC (12.5 uM) reached, and solvent DMSO added as the control group. Then, cultures in both groups were cultivated for another 2 h at 180 rpm. The microbial was centrifuged at 12,000 rpm for 10 min, washed twice with Phosphate Buffer Saline (PBS) buffer (Thermo Fisher Scientific, Ohio, USA), then kept at -80 °C until further protein extraction. The cell pellets were suspended in RIPA lysis buffer (Thermo Fisher Scientific, Ohio, USA) with cOmplete protease inhibitor cocktail (Roche, Switzerland). And then subjected to three rounds of homogenization. After centrifuged at 12,000 rpm for 20 min at 4 °C, the supernatants were collected for protein concentration determination with BCA protein assay kit (Beyotime Biotechnology, Shanghai, China). 100 μg protein was prepared and reduced with 10 mM DTT, followed by alkylation using 50 mM iodoacetamide. Following by desalting, the proteins were digested with trypsin overnight.

### Nano LC–MS/MS

Proteins were resuspended in 30 μL 0.1% formic acid (Thermo Scientific Pierce, Ohio, USA), 1 μg protein was injected onto an LC system consisting of an UltiMate 3000 RSLC nano system and a C18 precolumn, followed by separation using a C18 tip column (75 μm × 250 mm, Acclaim PepMap RSLC, 2 μm). The column was coupled to Q Exactive Plus mass spectrometer (Thermo Fisher Scientific, Ohio, USA) equipped with the Nano spray ionization (NSI) interface. MS1 scans were covered a mass range of 300–1500 m/z with a resolution of 70,000 and the MS2 spectra were acquired at a resolution of 17,500, collected for maximally 50 ms.

### Bioinformatics analysis

Proteome Discoverer 2.4 based with Sequest HT was used for protein identification and quantification, conducted against the Uniprot proteome of *Staphylococcus aureus* (Strain: NCTC 8325). Up-regulated proteins and down-regulated proteins were decided by calculating the *p*-value < 0.05 in at least two replicates and a 1.5-fold cut-off value. GO annotation applied for differentially expressed proteins was done with the DAVID database. The PPI networks were analyzed using the STRING database.

### Statistical analysis

Data were analyzed by Student’s t-tests using SPSS software (version 17.0, Chicago, IL, USA). *P* values < 0.05 were regarded as statistically significant.

## Supplementary Information

**Additional file 1.**

**Additional file 2.**

**Additional file 3.**

## Data Availability

All data generated or analyzed during this study are included in this published article. The datasets generated and/or analysed during the current study are available in the PRIDE repository with the dataset identifier PXD031706 (Project https://doi.org/10.6019/PXD031706). Project Webpage: http://www.ebi.ac.uk/pride/archive/projects/PXD031706 FTP Download: http://ftp.pride.ebi.ac.uk/pride/data/archive/2022/03/PXD031706

## Abbreviations

MRSA: Methicillin-resistant *Staphylococcus **aureus*
MSSA: Methicillin-sensitive *Staphylococcus **aureus*
MIC: Minimal inhibitory concentration
LPA: Lipoteichoic acid
LDL: Low-density lipoprotein
PBS: Phosphate Buffer Saline
OD: Optical density
GO: Gene Ontology
PPI: Protein–protein interaction
AST: Antimicrobial susceptibility testing
MS: Mass spectrometry
TSB: Tryptone soy broth
