# Supplementary material for: Antibacterial activities and action mode of anti-hyperlipidemic lomitapide against Staphylococcus aureus
Source: BMC Microbiol. 2022 Apr 26;22:114. doi: 10.1186/s12866-022-02535-9 (PMC9040290; doi:10.1186/s12866-022-02535-9)
Supplement: Supplementary file 1 — Additional file 1. [file 12866_2022_2535_MOESM1_ESM.docx]

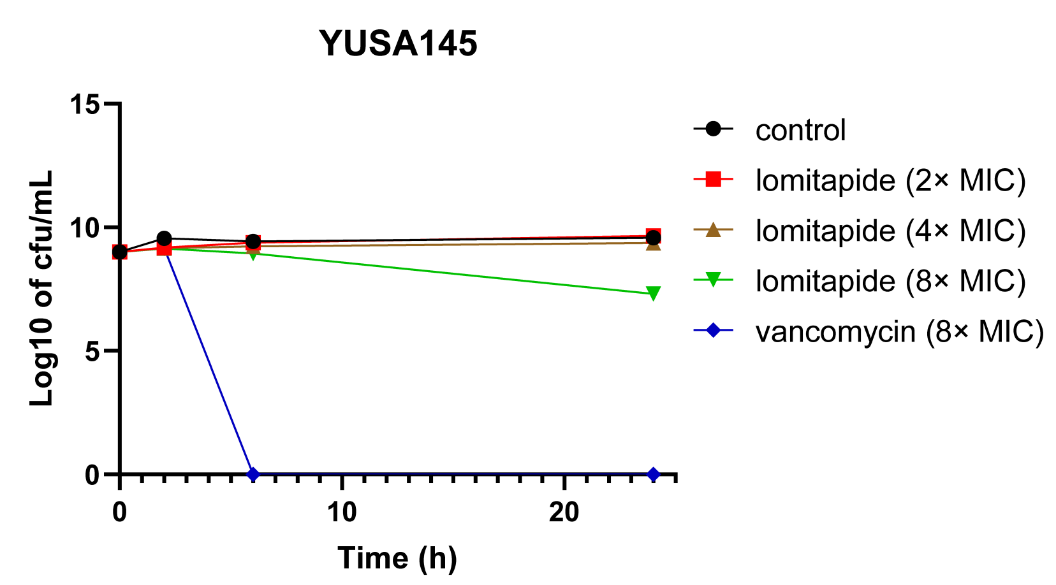


**Fig. S1** Time-kill assay of lomitapide against MRSA strain YUSA145. The bactericidal effect of lomitapide were assayed at various concentrations, including 2× MIC, 4× MIC, 8× MIC. The MIC of YUSA145 for lomitapide is 25 μM. Vancomycin was used at 8× MIC as a positive control. Bacteria CFU amount was measured at 0 h, 1 h, 3 h, and 24 h. Data are presented as means ± SD.
