# Supplementary material for: Antibacterial activities and action mode of anti-hyperlipidemic lomitapide against Staphylococcus aureus
Source: BMC Microbiol. 2022 Apr 26;22:114. doi: 10.1186/s12866-022-02535-9 (PMC9040290; doi:10.1186/s12866-022-02535-9)
Supplement: Supplementary file 2 — Additional file 2. [file 12866_2022_2535_MOESM2_ESM.docx]

**Table S1**. Antimicrobial susceptibility of clinical S. aureus isolates against lomitapide and commonly used antibiotics.

|  |  | **MIC (mg/L)** | | | |  |  |  |  | **MIC** |  |
| --- | --- | --- | --- | --- | --- | --- | --- | --- | --- | --- | --- |
|  | **Stain** | **Oxa** | **Cef** | **Van** | **LZD** | **Dap** |  | **Lomitapide** | | | |
| **MSSA** |  |  |  |  |  |  |  |  | | | |
|  | CHS25 | 0.5 | 0.25 | 1 | 4 | 2 |  | 25 μM/17.34 mg/L | | | |
|  | CHS26 | 0.5 | 0.25 | 1 | 4 | 2 |  | 12.5 μM/8.67 mg/L | | | |
|  | CHS30 | 0.5 | 1 | 1 | 2 | 2 |  | 25 μM/17.34 mg/L | | | |
|  | CHS101 | 0.5 | 0.5 | 1 | 2 | 2 |  | 25 μM/17.34 mg/L | | | |
|  | YUSA21 | 0.5 | 1 | 1 | 4 | 2 |  | 25 μM/17.34 mg/L | | | |
|  | YUSA80 | 0.5 | 1 | 1 | 4 | 2 |  | 12.5 μM/8.67 mg/L | | | |
|  | ATCC29213 | 0.25 | 0.5 | 1 | 4 | 2 |  | 12.5 μM/8.67 mg/L | | | |
|  | SA113 | 0.25 | 0.25 | 1 | 2 | 2 |  | 12.5 μM/8.67 mg/L | | | |
| **MRSA** |  |  |  |  |  |  |  |  | | | |
|  | CHS350 | >512 | >512 | 2 | 2 | 2 |  | 25 μM/17.34 mg/L | | | |
|  | CHS712 | >512 | >64 | 1 | 4 | 2 |  | 25 μM/17.34 mg/L | | | |
|  | CHS780 | >512 | >64 | 1 | 4 | 4 |  | 12.5 μM/8.67 mg/L | | | |
|  | YUSA139 | >64 | >64 | 1 | 4 | 4 |  | 25 μM/17.34 mg/L | | | |
|  | YUSA142 | >64 | >64 | 1 | 4 | 4 |  | 25 μM/17.34 mg/L | | | |
|  | YUSA145 | >64 | >64 | 1 | 4 | 4 |  | 25 μM/17.34 mg/L | | | |
|  | HaMRSA129 | >64 | >64 | 1 | 4 | 4 |  | 12.5 μM/8.67 mg/L | | | |
|  | HaMRSA19 | >64 | >64 | 1 | 4 | 4 |  | 25 μM/17.34 mg/L | | | |

*MSSA* methicillin-susceptible *Staphylococcus aureus*. *MRSA* methicillin-resistant *Staphylococcus aureus*. *Oxa* oxacillin. *Cef* ceftazidime. *Van* vancomycin. *LZD* linezolid. *Dap* daptomycin.

**Table S2**. the MIC distribution of lomitapide against clinical *E. faecalis, E. faecium, and S. agalactiae* isolates.

| Species | No. of isolates tested | MIC_50_ (μM) | MIC_90_ (μM) | MIC Range (μM) |
| --- | --- | --- | --- | --- |
| *S. epidermidis* | 10 | 12.5 | 25 | 12.5-25 |
| *E. faecalis* | 10 | 25 | 25 | 12.5-50 |
| *E. faecium* | 10 | 25 | 25 | 12.5-25 |
| *S. agalactiae* | 10 | 25 | 25 | 12.5-25 |

**Table S3**. the MICs of lomitapide against Gram-negative bacteria.

| Species | isolate | MIC  (μM) |
| --- | --- | --- |
|  |  |  |
| *Acinetobacter baumannii* | AB1 | ＞200 |
| *Klebsiella pneumoniae* | K2044 | ＞200 |
| *Escherichia coli* | ATCC25922 | ＞200 |
| *Pseudomonas aeruginosa* | ATCC27853 | ＞200 |

**Table S5**. Most enriched GO terms and KEGG pathway in differentially expressed proteins

| Category | Term | Description | Differentially expressed proteins |
| --- | --- | --- | --- |
| Gene Ontology_ Biological Process | GO:0007155 | cell adhesion | fibrinogen-binding protein SdrC, fibrinogen-binding protein SdrD, fibronectin binding protein B |
|  | GO:0006979 | response to oxidative stress | glutathione peroxidase, methionine sulfoxide reductase A, methionine sulfoxide reductase B |
|  | GO:0032784 | regulation of DNA-templated transcription, elongation | transcription antitermination protein, transcription elongation factor GreA |
| KEGG_PATHWAY | sao05150 | Staphylococcus aureus infection | D-alanine--poly(phosphoribitol) ligase subunit 1, D-alanine--poly(phosphoribitol) ligase subunit 2, MHC class II analog protein, fibrinogen-binding protein SdrC, fibrinogen-binding protein SdrD, immunoglobulin G-binding protein Sbi |
